# The impact of agricultural soil usage on activity and abundance of ammonifying bacteria in selected soils from Poland

**DOI:** 10.1186/s40064-016-2264-8

**Published:** 2016-05-10

**Authors:** Agnieszka Wolińska, Anna Szafranek-Nakonieczna, Artur Banach, Mieczysław Błaszczyk, Zofia Stępniewska

**Affiliations:** Department of Biochemistry and Environmental Chemistry, Institute of Biotechnology, The John Paul II Catholic University of Lublin, Konstantynów 1 I Str., 20-708 Lublin, Poland; Department of Microbial Biology, Warsaw University of Life Sciences, 159 Nowoursynowska, Str., 02-776 Warsaw, Poland

**Keywords:** Arginine ammonification, Ammonifying bacteria, Agricultural soil usage, Nitrogen forms, pH

## Abstract

The aim of the study was to demonstrate the impact of soil agricultural usage on the abundance of ammonifying bacteria (AB) and their activity, expressed as arginine ammonification (AA). Five agriculturally exploited types of soils (FAO): *Haplic Luvisol*, *Brunic Arenosol*, *Mollic Gleysol*, *Eutric Fluvisol*, and *Rendzina Leptosol* were studied. The controls were non-agricultural soils of the same type located in close proximity to agricultural sites. The tested soils varied in terms of pH (4.18–7.08), total carbon (8.39–34.90 g kg^−1^), easily degradable carbon content (0.46–1.11 g kg^−1^), moisture (5.20–13.50 %), and nitrogen forms (mg kg^−1^): 1.68–27.17, 0.036–0.862, 0.012–3.389 for nitrate nitrogen, nitrite nitrogen, and ammonia nitrogen, respectively. The AB abundance in agricultural soils ranged from 1.1 to 6.4 × 10^4^ cfu g^−1^, while in the controls it was significantly higher—from 2.0 to 110 × 10^4^ cfu g^−1^ of soil. Also, AA in the controls was three-times higher than in the agricultural soils. Strong associations between AA and the abundance of AB in the control (r = 0.954***) and agricultural soils (r = 0.833***) were proved. In the agricultural soils, the AB abundance and AA were influenced by pH (r = 0.746*** and r = 0.520***) and carbon content (r = 0.488*** and r = 0.391***). The AB abundance was also affected by easily degradable carbon (r = 0.517**) and nitrite nitrogen (r = 0.376*), whilst ammonium nitrogen influenced AA (r = 0.451*). Our results indicate that the abundance of AB and AA may be good indicators of soil biological conditions.

## Background

Nitrogen transformation in agro-ecosystems is indispensable to sustain the future crop production (Bhuyan et al. 2014). N-mineralization, a microbial process supplying mineral N to plants in terrestrial ecosystems (Doran 1987; Bach et al. 2012; Wang et al. 2013), is influenced by biomass inputs, microbial activities, and different abiotic factors such as microclimatic variations and land use patterns (Bhuyan et al. 2014). Land use change plays a very important role in regulating soil N mineralization and availability by altering soil biological, physical, and chemical properties (Marquard et al. 2009; Campos 2010). Thus, it is assumed that intensification of agricultural practices modifies the plant composition and soil characteristics that regulate the water content, nutrient availability (Rhoades and Coleman 1999), pH (Campos 2010), and carbon content (Wolińska et al. 2014). The study performed by Mäder et al. (2002) and Nourbakhsh and Alinejadian (2009) clearly indicated that intensive agriculture has increased crop yields but also posed severe environmental problems. A majority of them are associated with the high input of N fertilizers in intensive crop production (Deng and Tabatabai 2000; Marquard et al. 2009). As described by Nourbakhsh and Alinejadian (2009), N mineralization is a process of conversion of organic forms of N to ammonium (NH_4_^+^), which can be easily taken up by plants and thus play a vital role in plant growth (Doran 1987; Wang et al. 2013).

Our paper considers arginine ammonification activity as a biological function participating in the mineralization of organic N. Earlier reports also suggested that soil microorganisms catabolize arginine via different pathways, and NH_4_^+^ is a predominant end product (Nourbakhsh and Alinejadian 2009). Additionally, AA can be considered as an estimation of soil microbial-population size and their general activity (Alef and Kleiner 1987; Bonde et al. 2001; Nourbakhsh and Alinejadian 2009), and thus it is a convenient, inexpensive, sensitive, and relatively fast method recommended for estimation of microbial potential activity (Alef and Kleiner 1987; Kresović and Liĉina 2002).

It is known that each soil type has its own microflora that is affected positively or negatively by the pattern of soil usage, which is directly reflected in soil fertility (Tintor et al. 2009; Marinkovic et al. 2012; Kheyrodin et al. 2012). Among the huge diversity of microorganisms in the soil environment, chemolitho-autotrophic AB from the β subclass of *Proteobacteria* (Kowalchuk and Stephen 2001; Qiu et al. 2010) are ubiquitous and found in nearly all soil, freshwater, and marine environments (Horz et al. 2004; Qiu et al. 2010). Moreover, they are of great environmental importance, as NH_4_^+^ oxidation is the rate-limiting step of nitrification and is thus central to the global nitrogen cycle (Kowalchuk and Stephen 2001; Wielgosz et al. 2010). For these reasons, we suggest that AB might be an adequate indicator of soil biodegradation processes occurring in agricultural soils.

The fact that soil management strongly influences soil biodiversity, for example in agricultural ecosystems, was described by Valpassos et al. (2001) and Imaz et al. (2010). Moreover, Tscharntke et al. (2012) indicated that agricultural types of soil contain considerably smaller numbers of microorganisms (about 140–150 species per g of soil), in comparison with natural soils (1000s of species per g of soil). Also, the reduction in the number of bacterial phylogenetic groups affected by human agricultural activities is a cause of the small taxonomic group content in arable soils (Tscharntke et al. 2012). Finally, the lack of biodiversity is another consequence for agricultural soils not being able to become naturally regenerated, which may lead to their inability of regaining a satisfactory level of fertility (Wolińska et al. 2014). Additionally, the management practices used in many agro-ecosystems (e.g. monocultures, extensive use of tillage, chemical inputs) degrade the fragile web of community interactions between pests and their natural enemies and lead to increased pest and disease problems (Valpassos et al. 2001; Marquard et al. 2009; Kheyrodin et al. 2012). A question arises about what management practices adversely affect the functioning of soil ecosystems.

Consequently, we hypothesised that both AA and abundance of AB are strongly affected by land use and might be an adequate and easily measurable indicator of biological degradation processes. The particular objectives of the study were as follows: (1) quantification of soil N forms and AB abundance in five different arable and non-cultivated soil types, and (2) determination of correlations between soil environmental factors influencing AB abundance and AA in agricultural and control soils. Our aim is strictly consistent with the recommendations of the European Environmental Agency (EEA) and the Commission of the European Communities (EC), European Union (EU), biodiversity strategy to 2020 and European project ENVASSO, strongly promoting the description of soil microbiological degradation state.

## Methods

### Study site

The presented study was conducted as part of an ongoing long-term experiment (established in 1991) connected with organization of a database of Polish arable mineral soils (Bieganowski et al. 2013). The Bank of Soil Samples (BSS) was established through inspirations from Gliński, Ostrowski, Stępniewska, and Stępniewski (Gliński et al. 1991) to serve as a depository of catalogued soil samples stored dried and ready for use for soil research. Between 24 and 26 April 2014, 23 years after BSS organization, we returned to selected locations from the Lublin region in order to take arable soils for biological analysis. Precise locations of soils selected for the current study are shown in Table 1. It should be emphasized that the Lublin district is a representative region characterized by a great diversity of soil types and it is one of the largest and the most important agricultural areas in Poland (Wolińska et al. 2014).

**Table 1:** Location of agricultural soils with crop type and description of control sites (Lublin region)

Soil no.	Soil type (FAO)	Crop type (2014)	Village	Geographic coordinates	Control sites
1	*Haplic Luvisol*	Oat	Dęba	22°10′17.7″ 51°26′24.6″	30 year old meadow planted with fruit trees
2	*Haplic Luvisol*	Triticale	Pryszczowa Góra	22°27′10.3″ 51°24′3.8″	20 year old woodlots with birches
3	*Brunic Arenosol*	Triticale	Klementowice	22°06′54.2″ 51°21′52.2″	Unmoved meadow, wasteland
4	*Brunic Arenosol*	Oat	Łany	22°15′19.0″ 51°23′0.9″	20 year old field-woodlots
5	*Brunic Arenosol*	Oat	Markuszów	22°15′55.5″ 51°23′1.9″	20 year old field-woodlots
6	*Brunic Arenosol*	Field prepared for seeding	Rogalin	24°04′0.3″ 50°51′15.81	Meadow (mowed once a year)
7	*Brunic Arenosol*	Strawberries	Chrząchówek	22°07′29.9″ 51°25′5.5″	Unmoved meadow, wasteland
8	*Mollic Gleysol*	Wheat	Bałtów	22°01′25.5″ 51°29′15.3″	70 year old meadow (mowed once a year)
9	*Eutric Fluvisol*	Oat	Kośmin	21°59′10.1″ 51°33′47.7″	15 year old meadow (mowed once a year)
10	*Rendzina Leptosol*	Celeries	Siedliszcze	23°10′58.3″ 51°12′22.3″	40 year old meadow (mowed once a year)

### Soil characteristics

Soil samples were taken using Egner’s bow from the topsoil layer (0–20 cm) of agriculturally exploited sites (three replicates of 2 kg, consisting of about 50 samples taken from a 100 m^2^ area). Arable soils were collected from non-ploughed sites in order to avoid artifacts from ploughing perturbations (Wolińska et al. 2014). Consistently, control samples were taken from non-agriculturally cultivated and non-forested sites (at least 1-ha area) located in close vicinity to the basic soils and belonging to the same soil type (Table 1). As a control, old meadows or field-woodlots were selected. For the study, five types of soils were chosen: *Haplic Luvisol*, *Brunic Arenosol*, *Mollic Gleysol*, *Eutric Fluvisol*, and *Rendzina Leptosol*. Under laboratory conditions, each sample was passed through a 2.0-mm sieve to remove large pieces of rocks and plant material and stored at 4 °C prior analysis.

The moisture content was determined according to the ASTM D2216-10 norm (1999) by the gravimetric method (24 h, 105 °C).

The pH values were determined from a 2:1 soil suspension in distilled water using a multifunctional potential meter (Hach Lange) equipped with a glass electrode (pH E16M340). The measurements were taken in triplicate after stabilisation of the readings.

Total carbon (TC) was determined using an automatic carbon analyser TOC-V_CSH_ SSM 5000A (Shimadzu, Japan). Soil samples (150 mg) were pulverized, dried prior to analysis, and then combusted at 900 °C in a column containing a platinum and cobalt oxide catalyst (Wolińska et al. 2014). Under these conditions, all carbon compounds were converted into carbon dioxide and detected by an infrared detector (NDIR). Each TC recording was realized in triplicate.

Permanganate oxidizable carbon (POXC), i.e. an equivalent of carbon easily available to microorganisms, was determined according to the Weil et al. (2003) method based on the carbon oxidation by permanganate resulting in bleaching of a purple solution. Prior to the analysis, a standard curve was prepared using 0.2 KMnO_4_ in a 1-M CaCl_2_ (pH 7.2) stock solution. Absorbance was measured at 550 nm using distilled water as a background (UV-1800 Shimadzu). For the calculation of the POXC content in the soil sample, the following formula was used (Weil et al. 2003):

$$\begin{aligned} {\text{Active}}\,{\text{C}}\,\left( {{\text{mg}}\,{\text{kg}}^{ - 1} } \right) & = [0.02\,{\text{mol}}/{\text{l}}{-}(a + b \times {\text{absorbance}})] \times (9000\,{\text{ mg}}\,{\text{C/mol}}) \\ & \quad \times (0.02\,{\text{l}}\,{\text{solution}}/0.0025\,{\text{kg}}\,{\text{soil}}) \\ \end{aligned}$$

where 0.02 mol/l is the initial solution concentration, *a* and *b* are the intercept and the slope of the standard curve, respectively, 9000 is the amount of C in mg oxidized by 1 mol of MnO_4_, 0.02 l is the volume of the KMnO_4_ solution that has reacted, and 0.0025 is the amount of the soil sample in kg.

The concentrations of NH_4_-N, NO_3_-N, and NO_2_-N were determined colorimetrically using an Auto Analyser 3 System (Bran+Luebbe, Germany). Prior to analysis, the examined nutrients were extracted using deionised water (35 g fresh soil and 100 ml water). NH_4_-N and NO_3_-N were measured using, respectively, hydrazine sulphate (Kamphake et al. 1967) and salicylate (Grasshoff and Johannsen 1972) as a colour marker. The NO_2_-N analysis was based on the latter method excluding hydrazine sulphate. The results obtained were corrected for the amount of the soil sample and expressed as mg per kg of fresh soil.

### Arginine ammonification

Potential AA was quantified using the method proposed by Alef and Kleiner (1987) with modification by Wyczółkowski and Dąbek-Szreniawska (2005). Two portions equivalent to 5 g oven dry soil were taken. One portion was incubated for 4 h at 30 °C in an incubator after addition of 2 ml of 11.5 M arginine solution, whereas the other was used as a control where distilled water instead of the arginine solution was applied. Then, the soils were extracted with 18 ml 2 M KCl by 30 min shaking and filtered through Whatman 42 filter paper. Five millilitres of 2-M KCl, 2 ml of 0.12-M sodium phenolate, 1 ml of 0.17-mM nitroprusside sodium dehydrate, and 1 ml of sodium hypochlorite were added subsequently to 1 ml of the supernatants and incubated for 30 min in darkness. After that, absorbance was determined at 630 nm (UV-1800 Shimadzu). The AA rate was given in micrograms of NH_4_-N liberated per gram dry weight per hour.

### Abundance of ammonifiers

The soil samples (5 g each) were suspended separately in 50 ml 0.85 % NaCl. Then, each sample was prepared in tenfold dilution series and each dilution (1 ml) was inoculated into triplicate broth culture tubes for incubation (7 days, 26 °C), as described by Sutton (2010). For AB growth, 1 % water-peptone medium (g l^−1^: casein 10.0; NaCl 5.0; Na_2_HPO_4_ 1.5; KH_2_PO_4_ 9.0) was applied (Wolińska et al. 2013). Following 1-week incubation, Nessler reagent (0.5 ml) was added into each tube in order to detect ammonia presence/absence based on the colour reaction. All tubes in the three series were examined for colour reaction, and the specific patterns of growth in the tubes were scored against a most probable number (MPN) table for a three-replicate design from the US Food and Drugs Administration’s Bacterial Analytical Manual (Sutton 2010). The patterns of growth were read from the aforementioned table to provide MPN and a 95 % confidence interval (Sutton 2010). The populations of microbes were expressed as an MPN per g dry soil.

### Statistical analysis

Data were analysed using analysis of variance (ANOVA) for the randomized complete block. Mean separations were made for significant effects at *p* < 0.05 using the LSD post hoc test (Wolińska et al. 2014). Spearman’s rho correlation coefficient between chemical and biological soils properties was also determined. All statistical analyses were carried out using Statistica 9.0 (Statsoft Ltd., UK) software.

## Results and discussion

### Chemical soil characteristics

The major chemical characteristics of the studied soils are shown in Table 2. It was observed that the agricultural and control soils strongly differed in terms of moisture, pH, TC, POXC, and N forms. This differentiation confirms the fact that soil management is a crucial factor influencing not only soil biology but also soil chemical features. The agricultural soils were characterized by substantially lower pH values than the non-cultivated soils. pH in the arable soils ranged from 4.18 to 6.98; however, values similar to neutral pH were noted only in two cases (soil Nos. 3 and 6). In a majority of the investigated agricultural soils, pH oscillated in the range of acidic conditions (4.18 ± 0.05–5.58 ± 0.06). Similarly to pH, a decrease in the TC content in arable soils was observed and it oscillated between 8.30 ± 0.09 and 19.60 ± 0.05 g kg^−1^, whilst in the control soils the TC values were significantly higher and varied between 14.00 ± 0.05 and 34.90 ± 0.11 g kg^−1^ (*p* < 0.01). Nevertheless, the amount of carbon easily available to microorganisms, expressed as POXC, was even lower and ranged from 0.496 ± 0.07 to 0.760 ± 0.05 g kg^−1^ and from 0.546 ± 0.19 to 1.110 ± 0.03 g kg^−1^ for the agricultural and control soils, respectively. Generally, the pool of POXC accounted for only 4.0–6.0 % of TC. Guo et al. (2010), Swędrzyńska et al. (2013), and Wolińska et al. (2014) proved that systematic agricultural practices contribute to a significant decrease in pH values towards acidification, which is one of the causes of soil degradation. Also Dec (2014) reported that agricultural soils are characterized by a slightly acidic reaction (pH 6.3–6.5). Additionally, our observations might be supported by the findings of Valpassos et al. (2001), who reported that a no-tillage (controls) system showed the highest carbon content. Also Chan et al. (1992) and Gajic et al. (2006) assumed that continuous cropping and cultivation of many of the world’s soils has resulted in a substantial decline in the TC content.

**Table 2:** Chemical characteristics of agricultural (AGRIC) and control (CON) soils (±SD)

Soil no.	Land use	Moisture (%)	pH (H_2_O)	TC (g kg^−1^)	POXC (g kg^−1^)	NH_4_-N (mg kg^−1^)	NO_3_-N (mg kg^−1^)	NO_2_-N (mg kg^−1^)
1	AGRIC	8.20 ± 0.20	5.23 ± 0.06	9.80 ± 0.002	0.716 ± 0.01	0.012 ± 0.06	9.34 ± 0.08	0.110 ± 0.03
	CON	9.76 ± 0.11	6.27 ± 0.005	17.6 ± 0.12	0.546 ± 0.19	0.089 ± 0.05	1.68 ± 0.01	0.170 ± 0.01
2	AGRIC	9.30 ± 0.10	4.66 ± 0.02	12.3 ± 0.04	0.460 ± 0.03	0.017 ± 0.01	7.37 ± 0.05	0.082 ± 0.01
	CON	11.16 ± 0.11	5.02 ± 0.02	14.0 ± 0.05	0.571 ± 0.01	0.041 ± 0.01	5.84 ± 0.03	0.103 ± 0.02
3	AGRIC	12.56 ± 0.06	6.98 ± 0.02	19.60 ± 0.05	0.760 ± 0.05	0.431 ± 0.05	18.25 ± 0.06	0.100 ± 0.04
	CON	13.50 ± 0.10	7.08 ± 0.06	25.20 ± 0.14	0.946 ± 0.07	0.483 ± 0.02	7.57 ± 0.31	0.533 ± 0.03
4	AGRIC	6.60 ± 0.10	5.45 ± 0.04	10.10 ± 0.04	0.621 ± 0.03	0.072 ± 006	25.53 ± 0.18	0.116 ± 0.01
	CON	12.56 ± 0.06	5.58 ± 0.04	20.60 ± 0.19	0.828 ± 0.06	0.689 ± 0.09	10.18 ± 0.15	0.208 ± 0.02
5	AGRIC	9.23 ± 0.05	4.78 ± 0.006	8.30 ± 0.09	0.559 ± 0.06	0.014 ± 0.07	20.26 ± 0.07	0.096 ± 0.04
	CON	12.50 ± 0.08	5.58 ± 0.04	20.60 ± 0.19	0.828 ± 0.06	0.689 ± 0.09	10.20 ± 0.11	0.208 ± 0.02
6	AGRIC	12.13 ± 0.15	6.93 ± 0.006	9.70 ± 0.06	0.536 ± 0.08	0.05 ± 0.003	14.48 ± 0.04	0.036 ± 0.05
	CON	12.76 ± 0.11	6.99 ± 0.03	34.90 ± 0.11	1.110 ± 0.03	0.412 ± 0.08	5.41 ± 0.14	0.866 ± 0.03
7	AGRIC	5.66 ± 0.11	5.13 ± 0.006	8.80 ± 0.06	0.507 ± 0.09	0.018 ± 0.09	4.96 ± 0.06	0.080 ± 0.02
	CON	7.10 ± 0.17	5.40 ± 0.006	14.20 ± 0.11	0.575 ± 0.02	0.192 ± 0.08	1.75 ± 0.06	0.141 ± 0.01
8	AGRIC	5.80 ± 0.16	4.74 ± 0.02	9.10 ± 0.05	0.526 ± 0.01	0.128 ± 0.04	21.90 ± 0.02	0.091 ± 0.01
	CON	10.40 ± 0.17	6.25 ± 0.03	18.00 ± 0.13	0.795 ± 0.05	4.938 ± 0.08	6.75 ± 0.05	0.102 ± 0.01
9	AGRIC	5.20 ± 0.17	4.18 ± 0.05	9.80 ± 0.07	0.496 ± 0.07	0.140 ± 0.04	2.99 ± 0.03	0.105 ± 0.02
	CON	8.86 ± 0.11	5.64 ± 0.06	12.30 ± 0.08	0.621 ± 0.04	0.275 ± 0.03	2.19 ± 0.05	0.130 ± 0.01
10	AGRIC	10.86 ± 0.11	5.58 ± 0.06	9.70 ± 0.06	0.611 ± 0.05	0.049 ± 0.01	27.17 ± 0.14	0.095 ± 0.01
	CON	12.51 ± 0.17	5.76 ± 0.01	15.90 ± 0.12	0.694 ± 0.07	3.389 ± 0.06	10.12 ± 0.07	0.862 ± 0.07

The dominant form of N was the nitrate nitrogen (NO_3_-N); however, its level in the cultivated soils was substantially higher than in the controls and ranged between 2.99 ± 0.03 and 27.17 ± 0.14 mg kg^−1^; in turn, in the controls it was lower by 44–62.5 % and remained in the range of 1.68 ± 0.01–10.20 ± 0.11 mg kg^−1^. The dominance of nitrate nitrogen in the agricultural soils, especially during the spring season (soils were extracted in April 2014), can be explained by the fact that agricultural practices such as fertilization, particularly those performed after winter time (early spring), stimulate aerobic N transformation, resulting in nitrification of NH_4_-N (Campos 2010), and indirectly influence microbial activity. Booth et al. (2006) mentioned that NO_3_-N production and consumption in tilled soils are mediated more by TC and less than by the direct effects of disturbance; this is because as labile carbon is respired away, heterotrophs become less competitive and nitrifiers more competitive for NH_4_-N, resulting in increases in the size of the NO_3_-N pool. Two other forms of nitrogen (NH_4_-N and NO_2_-N) achieved the following values in the agricultural managed soils: 0.012 ± 0.06–0.431 ± 0.05 and 0.036 ± 0.05–0.116 ± 0.01 mg kg^−1^ for NH_4_-N and NO_2_-N, respectively. In contrast, the amounts of ammonium and nitrite nitrogen in the non-cultivated soils were even threefold–tenfold higher than those noted in the agricultural soils, and oscillated between 0.041 ± 0.01 and 4.938 ± 0.08 mg kg^−1^ in the case of NH_4_-N and from 0.102 ± 0.01 to 0.866 ± 0.03 mg kg^−1^ for NO_2_-N. Our results are in agreement with other studies showing variations in inorganic nitrogen, depending on land use (Banach et al. 2009; Campos 2010). However, the investigations by Campos (2010) were not strictly connected with arable soils, which were the subject in the current study, but related to tropical cloud forest, grassland, and coffee crop. Undoubtedly, the higher N richness noted in the cultivated soils is associated with continuous nitrogen fertilization thereof. This is consistent with the results of Li and Lang (2014) indicating a similar trend noted in uncultivated and cultivated black soil. Comparable findings were presented by Zhang et al. (2013) for woodland and agricultural soils. It should also be pointed that a high level of N fertilization can drive soil acidification (Guo et al. 2010) and modify the population numbers of soil microorganisms and availability of nutrients (Wolińska et al. 2014), which is the cause of differentiation of biological activity as an effect of agricultural management. Bach et al. (2012) indicated that grassland restoration on clay loam soils increases microbial biomass C and N, improves soil structure, and promotes soil C accrual.

### Arginine ammonification level

**Fig. 1:**
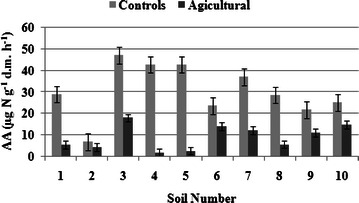
The content of NH_4_-N produced by arginine ammonification in agricultural and control soils. Average values with standard error are presented

The responses of AA to different soil management are presented in Fig. 1. Consistent with our hypothesis, there was a strong decline in AA as an effect of human agricultural practices, which confirms the fact that AA is a sensitive factor of biodegradation processes in cultivated soils. AA in the arable soils ranged from 1.86 to 17.98 µg N g^−1^ d.m. h^−1^, whereas in the control soils they were ca. twofold–fourfold higher and amounted to 6.89–47.02 µg N g^−1^ d.m. h^−1^. The maximum level of ammonium-N mineralised from arginine (17.98 and 47.02 µg N g^−1^ d.m. h^−1^ for the arable and control soils, respectively) was observed in *Brunic Arenosol* (No. 3). This confirms the results of Lin and Brookes (1999) and Kresović and Liĉina (2002), who determined AA at a level of 0.1–17.1 µg N g^−1^ d.m. h^−1^ and 4.5–17.8 µg N g^−1^ d.m. h^−1^ in arable soils, respectively. In their analysis of 34 soils, Alef and Kleiner (1987) reported that the AA rate usually ranged from 0.51 to 13 µg N g^−1^ d.m. h^−1^, whereas Kaiser et al. (1992) suggested much lower rates. In their 27 soils, AA ranged from 0.03 to 2.71 µg N g^−1^ d.m. h^−1^. Since most heterotrophic bacteria are able to ammonify arginine, AA is proportional to the soil microbial biomass and may be used as an indicator of microbial activity (Alef and Kleiner 1987; Lin and Brookes 1999; Kresović and Liĉina 2002). The greater rate of N mineralization in non-cultivated soils has been attributed to higher availability of organic N as well as to higher microbial activity, as indicated by Zaman et al. (2004). The recorded values of the AA rate confirm a tendency towards mineral N immobilisation in the soil, which is usually unfavourable for crop plants, although—on the other hand—it reduces oxidation of NH_4_-N–NO_3_-N in the process of nitrification, thus limiting potential losses of nitrogen (Frąc and Jezierska-Tys 2009).

### Abundance of ammonifying bacteria

**Fig. 2:**
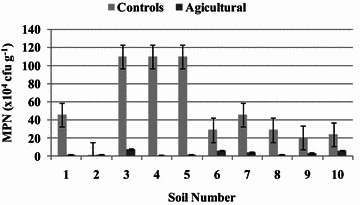
MPN of ammonifying bacteria in agricultural and control soils. Average values with standard error are presented

The results obtained (Fig. 2) indicate that the highest mean number of AB amounting to 21–110 × 10^4^ cfu g^−1^ was recorded for the control soils. A strong decrease in the AB abundance as an effect of agricultural management was reported in the case of the arable soils where MPN ranged from 1.5 to 7.5 × 10^4^ cfu g^−1^. The AB abundance in the control soils was by 93–98 % higher than in the arable soil samples. This indicates that systematic agricultural treatments contribute to a significant decrease in AB abundance, which is one of the causes of soil degradation, and that agricultural usage is a significant determinant of soil biological features.

Other papers demonstrated that AB abundance amounted to 0.17 × 10^3^ cfu g^−1^ in agricultural sandy loam with fungicide contamination (El-Imam and Machido 2012), 12 × 10^2^ cfu g^−1^ in apple orchard soil (Liu et al. 2013), and 6.0 × 10^4^ cfu g^−1^ in rice cropped soil (Li et al. 2008). In turn, Wolińska et al. (2013) estimated AB abundance in *Mollic Gleysol*, *Haplic Luvisol*, and *Eutric Fluvisol* at a level of 1.8–2 × 10^5^ cfu g^−1^. Investigations performed by Marinkovic et al. (2012) indicated that the highest abundance of ammonifiers was obtained in the case of chernozem, as well as in cambisol and solonetz, while the lowest number was found in fluvisol (8.13 × 10^6^ cfu g^−1^). The microbial population and its activity are known to depend greatly on soil pH (Guo et al. 2010; Swędrzyńska et al. 2013; Dec 2014; Wolińska et al. 2014), i.e. low pH values significantly decrease microbial activity, and AB abundance was lower in agricultural soils (Wielgosz et al. 2010; Liu et al. 2013; Bhuyan et al. 2014). However, to our knowledge, comparative studies of cultivated and uncultivated soils with regard to the MPN of AB are still limited. Important is also the fact that both AA as the abundance of AB displayed an analogical trend in relation to soil management practices. The control samples (Nos. 1, 3, 4, 5, 7) with the highest values of AA were simultaneously the most abandoned sites by AB and, vice versa, soil samples where AA reached a low level (i.e. No. 2) were characterized by a decrease in the AB number. This confirms that both AA and abundance of AB are closely related factors with similar sensitivity to human agricultural practices and thus might be recommended as indicators of soil biological degradation processes.

### Relationship between soil chemical and biological factors

The results obtained allowed identification of numerous relationships between soil chemical and biological parameters, with emphasis on the correlation between AA and the abundance of AB (Table 3). The correlations varied from the pattern of land use, which also confirmed the fact that environmental management is a strong determinant of soil biological and chemical features. Results of the regression analysis showed a linear relationship between pH and soil moisture (r = 0.788*** and 0.563**), pH and TC (r = 0.540* and r = 0.748***), and moisture and TC (r = 0.542** and 0.396*) for the agricultural and control soils, respectively. Analogously, a significant positive relationship between POXC, pH, and TC was also found both for the cultivated and control soils, as confirmed by the high values of Spearman’s rho correlation coefficient (Table 3). The relationship between NO_3_-N and chemical parameters is significant only in the agricultural soils in relation to moisture and POXC (r = 0.381* and r = 0.388*). On the contrary, the content of nitrate nitrogen in the control samples significantly influenced AA (r = 0.432*) and the abundance of AB (r = 0.431*). Similarly, a significant effect of NO_2_-N and NH_4_-N with regard to soil chemistry was noted only in the control soils (Table 3). In the cultivated soils, AA was significantly influenced by the following factors: pH (r = 0.520***), TC (r = 0.391***), and NH_4_-N (0.451*), whereas in the controls positive correlations were determined in the case of NO_3_-N and NH_4_-N, with the r coefficient amounting to 0.432* and 0.811***, respectively, and with soil moisture. The AB abundance in the agriculturally managed soils depended significantly on moisture (r = 0.570**), pH (r = 746***), TC (r = 0.488***), and POXC (r = 0.517**). In contrast, the effects of these chemical factors in the control soils were insignificant (*p* > 0.05), with one exception of POXC, for which a positive correlation was determined (r = 0.437*). Moreover, in the agricultural soils, a significant effect of NO_2_-N on the MPN of AB was found (r = 0.376*), whilst the AB abundance in the control was strongly dependent on the NH_4_-N content (r = 0.847***). In this context, it is worth emphasizing that AB are particularly active only if there is a high level of organic compounds in the soil and in the case of lack of mineral fertilization, as is the case in uncultivated soils. This is also related to the microbial life economics: if AB do not necessarily run the ammonification process, they use mineral nitrogen only. Finally, both in the agricultural and control soils, significant relationships between AA and the abundance of AB were determined to be r = 0.833*** and r = 0.954***. This interrelationship suggests that both parameters can be used as indicators of the soil biological degradation phenomenon.

**Table 3:** Correlations among investigated factors determined for agricultural (n = 30) and control soils (n = 30)

	pH	TC	POXC	NO_3_-N	NO_2_-N	NH_4_-N	AA	AB
Agricultural soil								
Moisture	0.788***	0.542**	0.606***	0.381*	ns	ns	ns	0.570**
pH		0.540*	0.657	ns	ns	ns	0.520***	0.746***
TC			0.702***	ns	ns	ns	0.391***	0.488***
POXC				0.388*	ns	ns	ns	0.517**
NO_3_-N					0.921***	0.574***	ns	ns
NO_2_-N						0.486**	ns	0.376*
NH_4_-N							0.451*	ns
AA								0.833***
Control soil								
Moisture	0.563**	0.396*	ns	ns	0.508**	ns	0.537*	ns
pH		0.748***	0.830***	ns	0.741***	0.362*	ns	ns
TC			0.948***	ns	0.936***	0.557**	ns	ns
POXC				ns	0.894***	0.649***	ns	0.437*
NO_3_-N					ns	0.547**	0.432*	0.431*
NO_2_-N						0.543*	ns	ns
NH_4_-N							0.811***	0.847***
AA								0.954***

*ns* no significance, *TC* total carbon, *POXC* permanganate oxidizable carbon, *AA* arginine ammonification, *AB* most probable number of ammonifying bacteria

* *p* < 0.05; ** *p* < 0.01; *** *p* < 0.001

Contrary to our results regarding agricultural soils, Tiquia et al. (2002) reported that AA was dependent neither on the change in TC (r^2^ = 0.04) nor N forms (r^2^ = 0.002), which remains in agreement to our finding but only with respect to the control sites. Rosenkranz et al. (2012) suggested that soil water content correlated positively with net ammonification rates and determined r = 0.25***. This is consistent with our study, as we also noted a positive effect of soil moisture on AA in the control soils (r = 0.537*). Alef and Kleiner (1987) and Lin and Brookes (1999) found no relationship between AA and pH, which is comparable to our data regarding the control soils (characterized by pH close to neutral); however, in the case of agricultural soils (characterized by lower pH than controls), we determined a significant effect of pH on AA (Table 3). The same effect connected with acidic pH was also suggested by Lin and Brookes (1999); however, they did not calculate Spearman’s rho correlation coefficient. Alef and Kleiner (1987) reported that the AA rate was closely correlated with the amount of soil microbial biomass. This is comparable to our results, as we noted a strong positive correlation (*p* < 0.001) between AA and the AB abundance, both in the agricultural as control soils. Additionally, in our work, there were highly significant correlations between NH_4_-N and AA (Table 3), which remains in agreement with the observations reported by Lin and Brookes (1999).

## Conclusions

Our data established that systematic agricultural practices significantly influenced soil biological and chemical properties. Comparison between the cultivated and control soils proved that agricultural exploited sites are biologically degraded, which was confirmed by the acidic pH, lower values of TC, and pool of POXC. The decreasing trend in the chemical factors was the cause of inhibition of AA and the AB abundance noted in arable soils. As demonstrated already in laboratory tests, the AA assay was quite sensitive and responded quickly to the mode of land use, similarly to the abundance of AB. The close relationship between these two factors was verified by the high values of Spearman’s rho correlation coefficient, i.e. r = 0.833*** and r = 0.954*** for the agricultural and control soils, respectively. Similarly, the hypothesis that both AA as AB abundance are strongly affected by human agricultural management and might be adequate and easily measurable indicators of the biological degradation process has been confirmed.
